# Pine mouth syndrome – an emerging food hypersensitivity?

**DOI:** 10.1186/2045-7022-1-S1-P25

**Published:** 2011-08-12

**Authors:** Ernest Kwegyir-Afful, Lowri DeJager, Tim Begley, Stefano Luccioli

**Affiliations:** 1 United States Food and Drug Administration, Center for Food Safety and Applied Nutrition, Riverdale, USA; 2 United States Food and Drug Administration, Center for Food Safety and Applied Nutrition, College Park, USA

## 

Between July 2008 and November 2010, the US Food and Drug Administration (FDA) received 197 consumer reports of prolonged taste disturbances following consumption of pine nuts. Most consumers consistently reported a delayed bitter or metallic taste with foods beginning 4 to 48 hours after pine nut consumption. This bitter taste recurred with any food consumed, lasting on average 5 to 10 days. Symptoms resolved in all cases without serious health consequences. To evaluate these complaints, a questionnaire was developed to address specific characteristics of the pine nuts consumed, associated symptoms and pertinent medical history, including allergies. In total, 130 of 197 consumers answered the questionnaire. All complainants were adults and 73.1% were female. The majority of the pine nuts were imported from Asia (China, 68.2%) and consumed in a raw state (75.2%). All consumers reported no rancid or foul taste with the pine nuts. Analysis of 15 different complaint samples found no evidence of pesticides or related contaminants; however, measurable concentrations of hexanal (0.2 - 4.1 mg/kg) – an indicator of lipid oxidation - were found. The majority of consumers were non-smokers and 30.8% reported one or more allergies: seasonal (20%), food (6.9%), drug (3.8%) and food intolerances (2.3%). Only 15.7% of consumers reported additional symptoms, such as tingling in the mouth, hives and/or gastrointestinal complaints, suggestive of a possible food allergy or intolerance. Except for a few cases in which oral allergy-like symptoms were noted in individuals with seasonal or tree nut allergies, there was no association between symptoms and an allergic condition. No other relevant medical information to explain the taste disturbances was found. The reported complaints are consistent with a previously described condition called pine mouth syndrome. Although the mechanism remains unknown, pine mouth syndrome should be recognized as an emerging food hypersensitivity in patients presenting with atypical oral complaints to pine nuts.

